# Supplementary material for: Shiga Toxin-Producing Escherichia coli (STEC) Associated with Calf Mortality in Uruguay
Source: Microorganisms. 2023 Jun 29;11(7):1704. doi: 10.3390/microorganisms11071704 (PMC10383947; doi:10.3390/microorganisms11071704)
Supplement: Supplementary file 1 [file microorganisms-11-01704-s001.zip › microorganisms-2430225-supplementary.pdf]

microorganisms

MDPI

Supplementary Materials: The following supporting information can be downloaded at: www.mdpi.com/xxx/s1, Figure S1: title; Table S1: title; Video S1: title.

Table S1. Origin of the animals.

Animal	Origin	Nº of isolates
1	spleen	8
	liver	8
2	lung	10
3	feces	9
	MLN	2
	lung	4
	intestine	7
4	kidney	2
	spleen	2
	cerebrospinal fluid	1
	urine	1
5	kidney	1
	liver	1
	heart	1
6	feces	6
7	lung	1
	intestine	2
8	bladder	8
	kidney	8
	liver	7
9	intestine	6
10	intestine	3
	lung	2
	MLN	1
11	intestine	4
12	feces	5
	intestine	5
13	brain	7
14	intestine	9
	MLN	10
15	feces	10
	brain	9
	intestine	10
	liver	10
	lung	10
	MLN	10
16	intestine	5
	MLN	3
	intestine	10
17	liver	3
	Total	*221*

MLN: mesenteric lymph node.

.

Table S2. Virulence profiles in *E. coli* isolates recovered from dead animals (n = 221)

Isolate	Origin	Virulence profile
1.1	spleen	*clpG/iucD*
1.2	spleen	*iucD/afa8E*
1.3	spleen	*clpG/iucD*
1.4	spleen	*f17A/iucD/papC*
1.5	spleen	*clpG/iucD*
1.6	spleen	*f17A/iucD/papC*
1.7	spleen	*f17A/iucD/papC/afa8E*
1.8	spleen	*f17A/iucD/papC*
1.9	liver	*clpG/iucD*
1.10	liver	*clpG/iucD*
1.11	liver	*clpG/iucD*
1.12	liver	*clpG/iucD*
1.13	liver	*clpG/iucD*
1.14	liver	*clpG/iucD*
1.15	liver	*clpG/iucD*
1.16	liver	*clpG/iucD*
2.1	lung	*iucD/afa8E*
2.2	lung	*iucD/afa8E*
2.3	lung	*iucD/afa8E*
2.4	lung	*iucD/afa8E*
2.5	lung	*iucD/afa8E*
2.6	lung	*iucD/afa8E*
2.7	lung	*iucD/afa8E*
2.8	lung	*iucD/afa8E*
2.9	lung	*iucD/afa8E*
2.10	lung	*iucD/afa8E*
3.1	feces	*eae/stx1/ehxA/cnf1/iucD*
3.2	feces	*eae/stx1/ehxA/cnf1/iucD*
3.3	feces	*eae/stx1/ehxA/cnf1/iucD*
3.4	feces	*eae/stx1/ehxA*
3.5	feces	*f17A*
3.6	feces	*f17A*
3.7	feces	*f17A*
3.8	feces	*clpG/afa8E*
3.9	feces	*clpG/afa8E*
3.10	feces	*papC*
3.11	lung	*papC*
3.12	lung	*papC*

3.13	lung	*papC*
3.14	intestine	*papC*
3.15	intestine	*papC*
3.16	intestine	*papC*
3.17	intestine	*papC*
3.18	intestine	*papC*
3.19	intestine	*eae/stx1/cnf1/iucD*
3.20	intestine	*papC*
3.21	MLN	*papC*
3.22	MLN	*papC*
4.1	kidney	*
4.2	kidney	*
4.3	spleen	*
4.4	spleen	*
4.5	brainespinal fluid	*
4.6	urine	*
5.1	kidney	*
5.2	liver	*
5.3	heart	*
6.1	feces	*iucD/papC*
6.2	feces	*eae/ehxA/iucD*
6.3	feces	*eae/ehxA/iucD*
6.4	feces	*eae/ehxA*
6.5	feces	*eae/ehxA/iucD*
6.6	feces	*eae/ehxA/iucD*
7.1	lung	*clpG/iucD/papC/afa8E*
7.2	intestine	*clpG/f17A/iucD/afa8E*
7.3	intestine	*clpG/f17A/iucD/papC/afa8E*
8.1	bladder	*
8.2	bladder	*
8.3	bladder	*
8.4	bladder	*f17A/f17GI*
8.5	bladder	*
8.6	bladder	*
8.7	bladder	*
8.8	bladder	*
8.9	kidney	*
8.10	kidney	*
8.11	kidney	*
8.12	kidney	*
8.13	kidney	*
8.14	kidney	*
8.15	kidney	*

8.16	kidney	*
8.17	liver	*f17A/f17GI/iucD*
8.18	liver	*iucD*
8.19	liver	*
8.20	liver	*
8.21	liver	*f17A/f17GI*
8.22	liver	*
8.23	liver	*f17A/f17GI/iucD*
9.1	intestine	*f17A/f17GI/iucD*
9.2	intestine	*
9.3	intestine	*
9.4	intestine	*
9.5	intestine	*eae/stx1/ehxA/iucD*
9.6	intestine	*
10.1	intestine	*
10.2	intestine	*eae/stx1/ehxA/iucD*
10.3	intestine	*iucD/afa8E*
10.4	lung	*eae/stx1/ehxA/iucD*
10.5	lung	*iucD/afa8E*
10.6	MLN	*eae/stx1/ehxA/iucD*
11.1	feces	*iucD/papC*
11.2	feces	*eae/stx1/ehxA/iucD*
11.3	feces	*eae/stx1/ehxA/iucD*
11.4	feces	*eae/stx1/ehxA/iucD*
12.1	feces	*f17A/f17GII/cnf2/cnf1/cdtIII/iucD*
12.2	feces	*f17A/f17GII/cnf2/cnf1/ cdtIII/iucD*
12.3	feces	*f17A/f17GII/iucD/papC*
12.4	feces	*f17A/f17GII/cnf2/cnf1/ cdtIII/iucD*
12.5	feces	*
12.6	intestine	*
12.7	intestine	*iucD/papC*
12.8	intestine	*iucD/papC*
12.9	intestine	*
12.10	intestine	*iucD/papC*
13.1	brain	*ehxA/cdtIII/papC*
13.2	brain	*ehxA*
13.3	brain	*ehxA*
13.4	brain	*ehxA*
13.5	brain	*ehxA*
13.6	brain	*papC*
13.7	brain	*ehxA*
14.1	intestine	*clpG/iucD/papC*
14.2	intestine	*clpG/iucD/papC*

14.3	intestine	*clpG/iucD/papC*
14.4	intestine	*clpG/iucD/papC*
14.5	intestine	*clpG/iucD/papC*
14.6	intestine	*clpG/iucD/papC*
14.7	intestine	*clpG/f17A/iucD/papC*
14.8	intestine	*clpG/f17A/f17GI/*
14.9	intestine	*clpG/iucD/papC*
14.10	MLN	*clpG/iucD/papC*
14.11	MLN	*clpG/iucD/papC*
14.12	MLN	*clpG/iucD/afa8E*
14.13	MLN	*clpG/f17A/iucD/afa8E*
14.14	MLN	*clpG/iucD/papC*
14.15	MLN	*clpG/iucD/papC*
14.16	MLN	*clpG/iucD/papC*
14.17	MLN	*clpG/iucD/papC*
14.18	MLN	*clpG/iucD/papC*
14.19	MLN	*clpG/iucD/papC*
15.1	feces	*eae/stx1/ehxA/iucD*
15.2	feces	*eae/stx1/ehxA/iucD*
15.3	feces	*eae/stx1/ehxA/iucD/afa8E*
15.4	feces	*eae/stx1/ehxA/iucD/afa8E*
15.5	feces	*eae/stx1/ehxA/iucD/afa8E*
15.6	feces	*eae/stx1/ehxA/iucD/afa8E*
15.7	feces	*eae/stx1/ehxA/iucD/afa8E*
15.8	feces	*eae/stx1/ehxA/iucD*
15.9	feces	*eae/stx1/ehxA/iucD*
15.10	feces	*eae/stx1/ehxA/iucD*
15.11	brain	*eae/stx1/ehxA/iucD*
15.12	brain	*eae/stx1/ehxA/iucD*
15.13	brain	*eae/stx1/ehxA/iucD/afa8E*
15.14	brain	*eae/stx1/ehxA/iucD/afa8E*
15.15	brain	*eae/stx1/iucD/afa8E*
15.16	brain	*eae/stx1/ehxA/iucD/afa8E*
15.17	brain	*eae/stx1/ehxA/iucD/afa8E*
15.18	brain	*iucD/afa8E*
15.19	brain	*eae/stx1/ehxA/iucD/afa8E*
15.20	intestine	*f17A/f17GII/iucD/afa8E*
15.21	intestine	*f17A/f17GII/iucD/afa8E*
15.22	intestine	*f17A/f17GII/iucD*
15.23	intestine	*f17A/f17GII/iucD/papC/afa8E*
15.24	intestine	*f17A/f17GII/iucD/afa8E*
15.25	intestine	*f17A/f17GII/iucD/afa8E*
15.26	intestine	*f17A/f17GII/iucD/afa8E*

15.27	intestine	*f17A/f17GII/iucD/afa8E*
15.28	intestine	*f17A/f17GII/iucD/afa8E*
15.29	intestine	*iucD/papC/afa8E*
15.30	liver	*eae/stx1/ehxA/iucD*
15.31	liver	*eae/stx1/ehxA/iucD*
15.32	liver	*eae/stx1/ehxA/iucD*
15.33	liver	*eae/stx1/ehxA/iucD*
15.34	liver	*eae/stx1/ehxA/iucD*
15.35	liver	*eae/stx1/ehxA/iucD*
15.36	liver	*eae/stx1/ehxA/iucD*
15.37	liver	*eae/stx1/ehxA/iucD*
15.38	liver	*eae/stx1/ehxA/iucD*
15.39	liver	*eae/stx1/ehxA/iucD*
15.40	lung	*eae/stx1/ehxA/iucD*
15.41	lung	*
15.42	lung	*stx1/ehxA/iucD*
15.43	lung	*eae/stx1/ehxA/iucD*
15.44	lung	*eae/stx1/ehxA/iucD*
15.45	lung	*eae/stx1/ehxA/iucD*
15.46	lung	*eae/stx1/ehxA/iucD*
15.47	lung	*eae/stx1/ehxA/iucD*
15.48	lung	*eae/stx1/ehxA/iucD*
15.49	lung	*eae/stx1/ehxA/iucD*
15.50	MLN	*iucD/afa8E*
15.51	MLN	*iucD/afa8E*
15.52	MLN	*f17A/f17GII/iucD*
15.53	MLN	*f17A/f17GII*
15.54	MLN	*f17A/f17GII*
15.55	MLN	*f17A/f17GII*
15.56	MLN	*f17A/f17GII*
15.57	MLN	*f17A/f17GII*
15.58	MLN	*f17A/f17GII/iucD/afa8E*
15.59	MLN	*f17A/f17GII/iucD/afa8E*
16.1	intestine	*f17A/f17GII/iucD/afa8E*
16.2	intestine	*iucD/afa8E*
16.3	intestine	*iucD/afa8E*
16.4	intestine	*f17A/f17GII/iucD/papC/afa8E*
16.5	intestine	*iucD/afa8E*
16.6	MLN	*iucD/afa8E*
16.7	MLN	*iucD/afa8E*
16.8	MLN	*iucD/afa8E*
16.9	feces	*eae/stx1/ehxA*
16.10	feces	*eae/stx1/ehxA*

16.11	feces	*eae/stx1/ehxA*
16.12	feces	*eae/stx1/ehxA*
16.13	feces	*eae/stx1/ehxA*
16.14	feces	*eae/stx1/ehxA*
16.15	feces	*eae/stx1/ehxA*
16.16	feces	*eae/stx1/ehxA*
16.17	feces	*eae/stx1/ehxA*
16.18	feces	*eae/stx1/ehxA*
17.1	liver	*
17.2	liver	*f17A/iucD/afa8E*
17.3	liver	*iucD/afa8E*

* Isolates in which none of the genes studied were detected. MLN: mesenteric lymph node.
